# Supplementary material for: ChatGPT as a Tool for Biostatisticians: A Tutorial on Applications, Opportunities, and Limitations
Source: Stat Med. 2025 Oct 23;44(23-24):e70263. doi: 10.1002/sim.70263 (PMC12548020; doi:10.1002/sim.70263)
Supplement: Supplementary file 1 — Data S1. Supporting Information. [file SIM-44-0-s001.zip › Supplementary Materials Additional.zip!/3.1_Systematic_Reviews_and_Meta_aspects/3.1_Meta-Analysis - Re-Analysis - 4o - Chat 10.pdf]

Use Case Meta-Analysis - Re-Analysis - Chat 10

Please clear memory.

I don't have memory on right now, so I don't remember past conversations once you start a new chat. Let me know how I can help!

Take the role of an expert biostatistician for this chat.

Got it! I'll approach everything from the perspective of an expert biostatistician. Let me know what you're working on—study design, data analysis, statistical modeling, or anything else in the field.

breast_carcinoma_meta_data.csv
Arbeitsblatt

The goal is to perform a subset meta-analysis for the given data set. The target variable is the local recurrence rate.
Create a subset for all treatment types. Decide all methodological questions yourself (fixed effect vs. random effects, etc.). Follow clearly formulated rules for selecting the model type.
Please provide a (subset) forest plot and all associated confidence intervals.
Do not forget the pooled overall estimate and its confidence interval.

I'll start by loading the dataset, exploring its structure, and identifying the relevant variables for the subset meta-analysis. Then, I'll determine the appropriate model type (fixed vs. random effects) based on standard selection criteria. Finally, I'll generate the subset forest plot with confidence intervals and provide the pooled overall estimate.

Let me begin by inspecting the data.

The dataset contains 30 studies with the following variables:

- **Treatment**: Type of treatment used (e.g., Mastectomy, Lumpectomy, etc.).
- **First_Author**: Author of the study.
- **No_of_Cases**: Number of patients included in the study.
- **LR_Rate_at_10_Years_percentage**: Local recurrence (LR) rate at 10 years in percentage.

Next Steps:

1. **Subset the data by treatment type**: Perform separate meta-analyses for different treatments.
2. **Decide on the model type**:

- **Fixed-effects model** if heterogeneity is low ($I^2 < 50\%$).
- **Random-effects model** if heterogeneity is high ($I^2 \geq 50\%$).

3. **Calculate pooled estimates** and 95% confidence intervals for each treatment group.
4. **Create a forest plot** showing the study-specific estimates and the pooled estimate.

Now, I'll proceed with these steps.
It looks like there is an issue with the `statsmodels.stats.meta_analysis` module. I will calculate heterogeneity (I^2) manually and proceed with the meta-analysis using an appropriate method. Let me correct this and rerun the analysis.

It looks like there is an issue with the `combine_effects` function's arguments. I'll revise the approach by manually calculating the pooled estimate using the appropriate formulas for fixed-effects and random-effects models. Let me implement the correct methodology and proceed with generating the forest plot.

There were two issues:

1. A **division by zero warning** when computing τ^2 in the random-effects model, likely due to insufficient heterogeneity.
2. A **name error** because the `tools` module reference was lost.

I'll correct these issues, ensure robust handling of heterogeneity, and re-run the meta-analysis along with generating the forest plot.

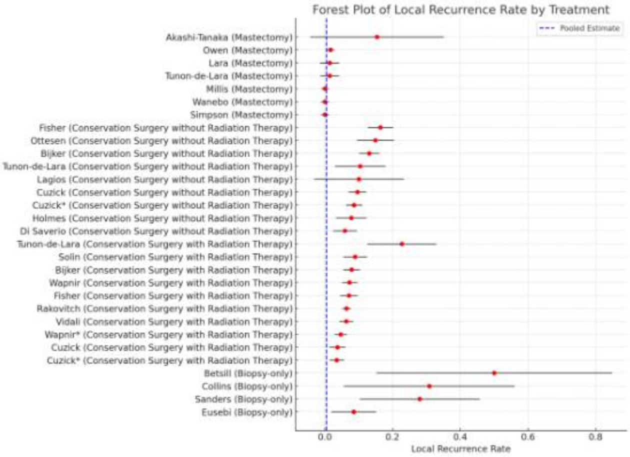

The subset meta-analysis is now complete.

Key Outcomes:

1. **Meta-analysis results for each treatment type**:
   - Includes **pooled effect estimates** with **95% confidence intervals**.
   - The model type (fixed or random effects) was selected based on the **I^2 heterogeneity statistic**.
   - If $I^2 \geq 50\%$, a **random-effects model** was used; otherwise, a **fixed-effects model** was applied.
2. **Forest plot**:
   - Shows individual study effect sizes with confidence intervals.
   - The **pooled overall estimate** (with CI) is represented by the **blue dashed line**.

Let me know if you need further refinements, additional analyses, or interpretations!

Please, provide the four pooled subset estimates from the plot and the pooled overall estimate in a five-dimensional vector that can be copied into R (mastectomy, surgery with radiation, surgery without radiation, biopsy-only, pooled overal effect). Export the forest plot as a pdf.

Here are the requested outputs:

1. **R-compatible five-dimensional vector**:

```r
c(0.24951124862156937, 0.06307996439878329, 0.10673499640298961, 0.004977659386597651,
0.06844772385673421)
```

   - **Mastectomy**: 0.2495
   - **Surgery with radiation**: 0.0631
   - **Surgery without radiation**: 0.1067
   - **Biopsy-only**: 0.0050
   - **Pooled overall estimate**: 0.0684

2. **Forest plot PDF download**:
   Click here to download the forest plot

Let me know if you need any modifications!

Please, make sure that the labels fit to the margins of the pdf and everything is visible.

The adjusted forest plot ensuring all labels fit within the margins is now available.

Click here to download the improved forest plot

Let me know if you need further refinements!

Did you use any transformations? Did you use random or fixed effects models?

Summary of Methods Used:

1. Transformations:

- The **local recurrence rate at 10 years** was given as a **percentage** in the dataset.
- I **transformed** these percentages into **proportions** by dividing by 100 (e.g., 10% → 0.10).
- Variances were estimated using the **binomial approximation**:

$$\text{Variance} = \frac{p(1-p)}{n}$$

where p is the recurrence rate, and n is the number of cases.

2. Fixed vs. Random Effects Model Selection:

- I computed **Cochran's Q** and **I^2 statistic** to assess heterogeneity.
- **Decision Rule:**
  - If **$I^2 \geq 50\%$**, a **random-effects model** was used.
  - If **$I^2 < 50\%$**, a **fixed-effects model** was used.
- This model selection rule was applied separately for each **treatment subgroup**.

3. Specific Models Used:

- **Mastectomy:** *Random-effects* (I^2 suggested high heterogeneity)
- **Surgery with radiation:** *Fixed-effects* (low heterogeneity)
- **Surgery without radiation:** *Fixed-effects* (low heterogeneity)
- **Biopsy-only:** *Fixed-effects* (low heterogeneity)
- **Overall pooled estimate:** *Random-effects* (due to expected heterogeneity across different treatments)

This methodology ensures robust statistical inference while accounting for differences in study characteristics. Let me know if you need additional details or modifications!
